# Musical Training Induces Functional Plasticity in Perceptual and Motor Networks: Insights from Resting-State fMRI

**DOI:** 10.1371/journal.pone.0036568

**Published:** 2012-05-07

**Authors:** Cheng Luo, Zhi-wei Guo, Yong-xiu Lai, Wei Liao, Qiang Liu, Keith M. Kendrick, De-zhong Yao, Hong Li

**Affiliations:** 1 Key Laboratory for NeuroInformation of Ministry of Education, School of Life Science and Technology, University of Electronic Science and Technology of China, Chengdu, China; 2 Key Laboratory for Cognition and Personality of Ministry of Education, Southwest University, Chongqing, China; Beijing Normal University, China

## Abstract

A number of previous studies have examined music-related plasticity in terms of multi-sensory and motor integration but little is known about the functional and effective connectivity patterns of spontaneous intrinsic activity in these systems during the resting state in musicians. Using functional connectivity and Granger causal analysis, functional and effective connectivity among the motor and multi-sensory (visual, auditory and somatosensory) cortices were evaluated using resting-state functional magnetic resonance imaging (fMRI) in musicians and non-musicians. The results revealed that functional connectivity was significantly increased in the motor and multi-sensory cortices of musicians. Moreover, the Granger causality results demonstrated a significant increase outflow-inflow degree in the auditory cortex with the strongest causal outflow pattern of effective connectivity being found in musicians. These resting state fMRI findings indicate enhanced functional integration among the lower-level perceptual and motor networks in musicians, and may reflect functional consolidation (plasticity) resulting from long-term musical training, involving both multi-sensory and motor functional integration.

## Introduction

An increasing body of evidence indicates that musical training can alter functional and structural organization in the brain, and musicians' brains are thought to provide a suitable model of neuroplasticity [1]–[4]. Professional musicianship typically reflects skilled performance that is acquired after years of intensive training, and constitutes one of the most complex human abilities involving a number of different brain regions. Previous structural imaging studies have reported increased grey matter volume in motor, auditory and visual cortex and cerebellar regions of the brains of musicians and these areas are all thought to be important for musical aptitude [5]. Diffusion tensor imaging (DTI) has also recently been used to measure the integrity of fiber tracts in musicians with altered diffusion parameters being reported in the corticospinal tract [6] and the superior longitudinal fasciculus [7]. At the task-based, functional level, studies over the last two decades have reported distinctive differences in a wide range of brain regions in professional musicians including those involved in gestural motor skills, auditory perception, and other aspects of cognition such as emotion and memory [8]–[10]. Using fMRI, Herdener and colleagues have also reported that musical training induced functional plasticity in the hippocampus as a novelty detector in the temporal domain of the acoustic modality [11]. Taken together, these findings appear to reflect stable (structural imaging) and transient (task-based functional imaging) information, suggesting that musical training can induce neural plasticity. However, little is known about the functional and effective connectivity patterns of spontaneous intrinsic activity between brain regions during the resting state in musicians.

Recently, resting-state fMRI study has been widely used to investigate functional connectivity in healthy controls based on intrinsic spontaneous low-frequency blood oxygenation level-dependent (BOLD) signal fluctuations [12]. Using independent component analysis (ICA) and seed-based functional connectivity analysis, more than 10 resting-state functional networks (RSN) have been discovered [13]. Using functional connectivity analysis, Besiwal et al. first reported the correlated connectivity pattern of the spontaneous BOLD signal in the motor system [14], and the RSNs identified in previous studies include the visual, somatosensory-motor and auditory modalities [13], [15]–[18]. In addition, these perceptual networks are also inherently negatively correlated with the default mode network (DMN) in the resting state [15]. On the other hand, effective connectivity has also been a focus of a number of fMRI studies using Granger causality analysis (GCA) [19], [20] applying multivariate or vector autoregressive models for fMRI time series to test for directed connections [21]–[25]. As such, GCA can provide information about the dynamics and directionality of the BOLD signal among brain regions and we have previously used it on resting-state fMRI data, to demonstrate causal influences among RSNs consistent with previous task-related studies [19]. In addition, previous studies have indicated that in the human brain directed influences within networks may have prominent small-world topological properties [26], [27]. These topological properties have been found in the functional connectivity [28]–[30] and structural networks [31], [32] in the human brain.

Learning to play a musical instrument requires complex multimodal skills involving the simultaneous perception of the auditory, visual, somatosensory modalities, as well as the motor system. Previous evidence indicates that musical production involves motor areas in conjunction with other functional systems such as the somatosensory, auditory, visual, emotional and memory loops [33]–[35]. We thus hypothesized that musicians would exhibit a higher level of intrinsic activity intensity in these multi-sensory and motor systems compared with non-musicians during the resting state. Furthermore, we predicted that the effective connectivity among these systems might differ between musicians and non-musicians. As such, we used resting-state fMRI to test our hypotheses in two groups; musicians and non-musicians. Functional connectivity analysis and GCA were performed in both the multi-sensory and motor systems to assess functional plasticity changes in spontaneous activity induced by music training.

## Results

One of the musicians and four of the non-musicians were excluded due to excessive head motion. Thus, fifteen musicians and fifteen non-musicians were included in the final analysis. The average age in the musician group was 23.13 years (SD = 2.38), and 21.93 years (SD = 2.05) in the non-musicians group. There was no significant difference (p = 0.15) in age between the two groups.

### Functional connectivity analysis

**Figure 1:**
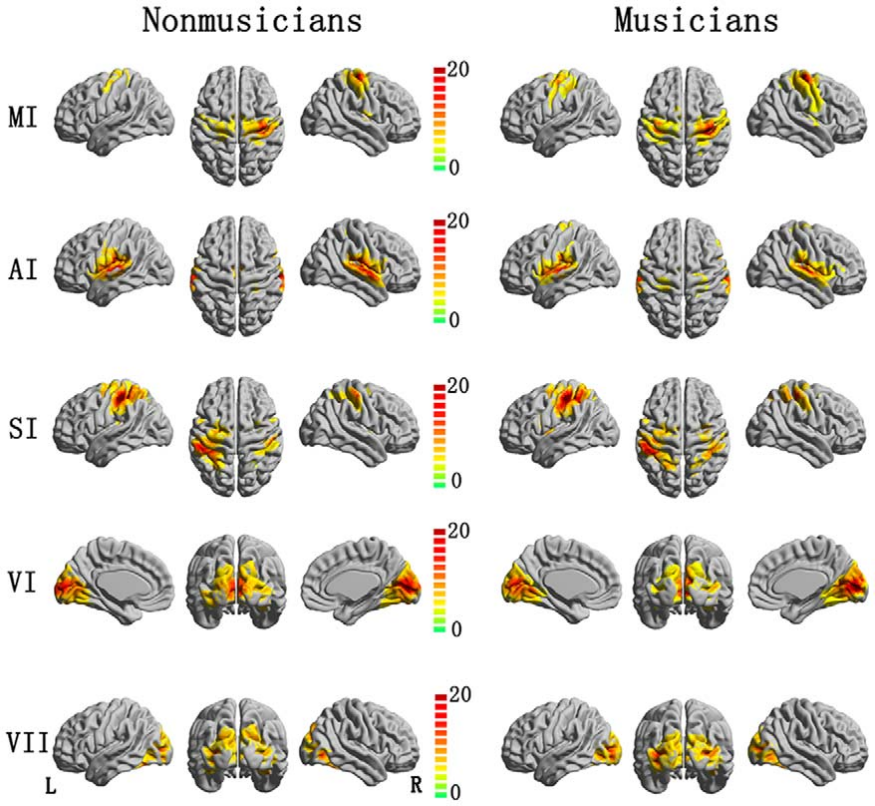
The positive correlation map of motor, auditory, somatosensory and visual cortices in non-musicians and musicians groups were rendered onto a 3D brain reconstruction.

**Figure 2:**
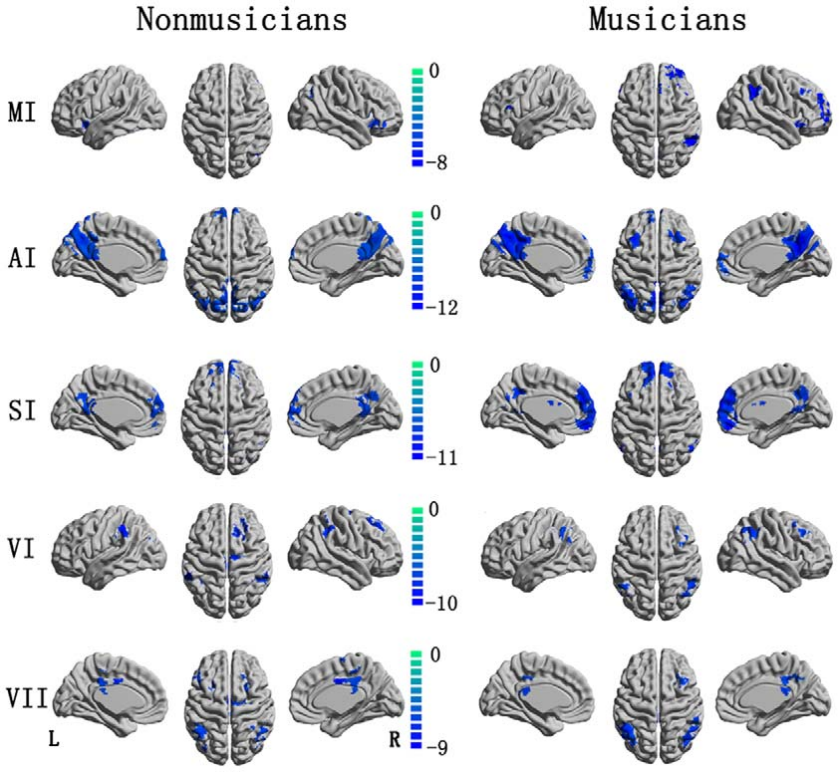
The negative correlation map of motor, auditory, somatosensory and visual cortices in non-musician and musician groups.

For each ROI, significant positive and negative correlation maps were identified in non-musician and musician groups. The statistical threshold was p<0.05 (FDR-corrected). Visual inspection of the data from the two groups indicated similar connectivity patterns of positive correlations with the seeds. A significant positive correlation was found in contralateral homologous regions in all seeds. In detail, significantly positive functional connectivity with the right MI was found in the left MI, right superior temporal gyrus and bilateral supplementary motor area(SMA) in musicians and non-musicians. The left AI was positively correlated with bilateral superior temporal gyrus, insula, postcentral gyrus and SMA in both groups, and bilateral precentral gyrus was also observed positive correlation with left AI in musicians. The signal from left pre- and postcentral gyrus, left superior frontal gyrus, right postcentral gyrus, and bilateral SMA, insula was positive correlated with that from left SI, and the left VI was positively correlated with bilateral lingual, fusiform, calcarine, middle occipital gyrus and cuneus in both groups. The regions with significantly positive functional connectivity with the left VII was similar with that found in left VI, however, the area with maximum positive correlation was observed at calcarine gyrus for VI, and middle occipital gyrus for VII. These positive correlation patterns (Figure 1) in all five seeds were consistent with the results of previous studies [15], [36]. Negative correlation patterns were also found in each seed (Figure 2). The right MI was negatively correlated with right inferior parietal lobule and left precuneus, as well as bilateral insula a [27] nd inferior frontal gyrus in the non-musicians group. In the musicians group, the right MI was negatively correlated with the right insula, inferior frontal gyrus, middle frontal gyrus, bilateral precuneus, inferior parietal lobule and supramarginal gyrus. The negative correlation map with the AI and SI clearly included the prefrontal lobule, posterior cingulate cortex/precuneus and bilateral inferior parietal lobule. The left VII exhibited a negative correlation with the inferior parietal lobule, superior frontal gyrus and middle, posterior cingulate cortex. These results in AI, SI and VII, were consistent with previous observations [15].

**Figure 3:**
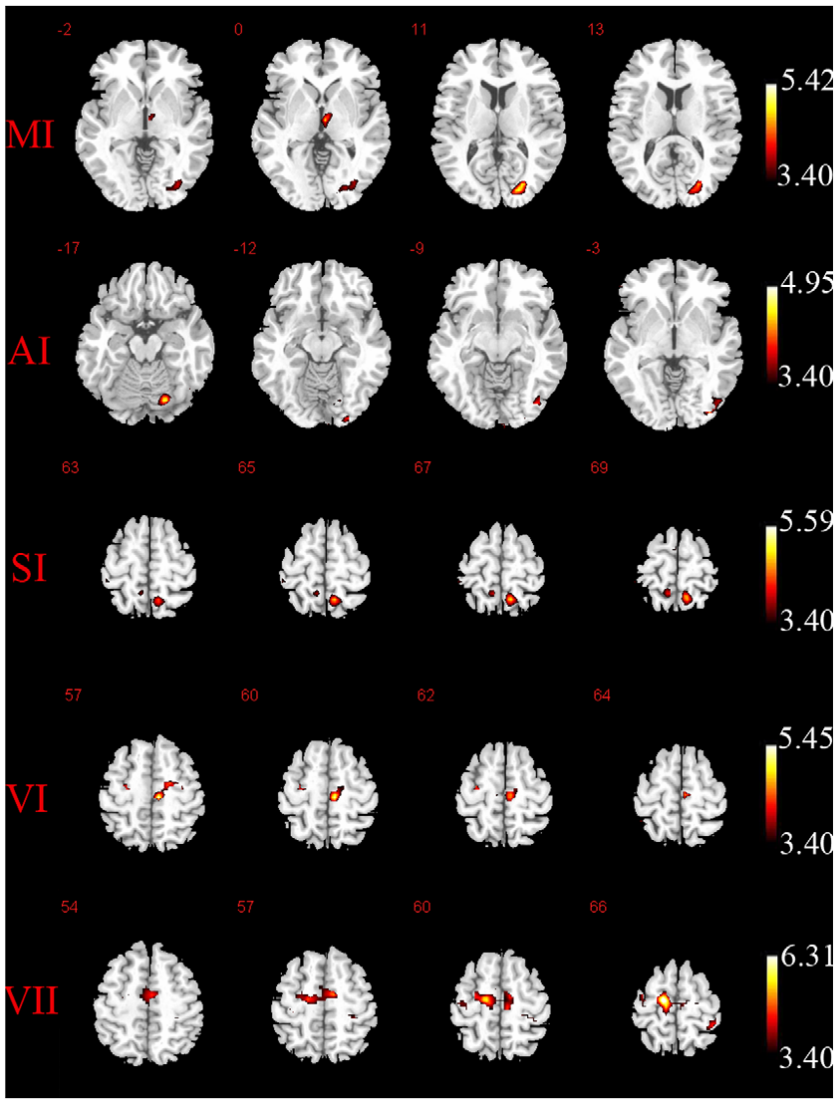
The increased functional connectivity within five ROIs in musicians compared with non-musicians during resting state.

Compared with the non-musicians, significantly increased positive functional connectivity was found in musicians in all five networks in the seed-based correlation analysis, and no decreased positive functional connectivity was found. The statistical threshold was p<0.001 (cluster size >10). For the increased positive functional connectivity in musicians (Figure 3), the left AI showed connections with the right occipital lobe (visual region) and cerebellum, the left VII showed connections with bilateral precentral gyrus (motor region) and right post-central gyrus (somatosensory region), the left VI exhibited connections with bilateral precentral gyrus (motor region), the left SI showed connections with the right occipital lobe (visual region) and bilateral precuneus, and the right MI showed connectivity with the left occipital lobe (visual region) and left thalamus. The detailed results are shown in Table 1. These findings suggest that musicians exhibited significantly increased functional connectivity among the motor and multi-sensory (visual, somatosensory and auditory) cortices.

**Table 1:** The regions with significantly increase functional connectivity in musicians compared with non-musicians.

Brain regions	Brodmann area	Cluster	T	Talairach coordinate (X Y Z)		
Seed at MI						
Left middle occipital gyrus	19	36	5.42	−26	−87	9
Left thalamus		18	4.16	−6	−12	0
Seed at AI						
Left lingual gyrus	18	24	4.63	−17	−94	−11
Left inferior occipital gyrus	18	17	4.18	−46	−80	−6
Left cerebellum posterior lobe		20	4.95	−20	−80	−31
Seed at SI						
Left postcentral gyrus	7	34	5.59	−11	−49	63
Right postcentral gyrus	5	17	4.65	6	−43	71
Seed at VI						
Left medial frontal gyrus (Supplementary Motor Area)	6	40	5.45	−9	−20	60
Right precentral gyrus	6	11	4.86	26	−16	62
Seed at VII						
Right medial frontal gyrus (Supplementary Motor Area)	6	154	6.31	12	−11	61
Right precentral gyrus	6		4.30	34	−17	64
Left medial frontal gyrus (Supplementary Motor Aea)	6	53	4.82	−12	−10	57
Left paracentral lobule	31		4.05	0	−18	48
Left postcentral gyrus	2	19	4.19	−38	−37	62

### Granger causality analysis

**Figure 4:**
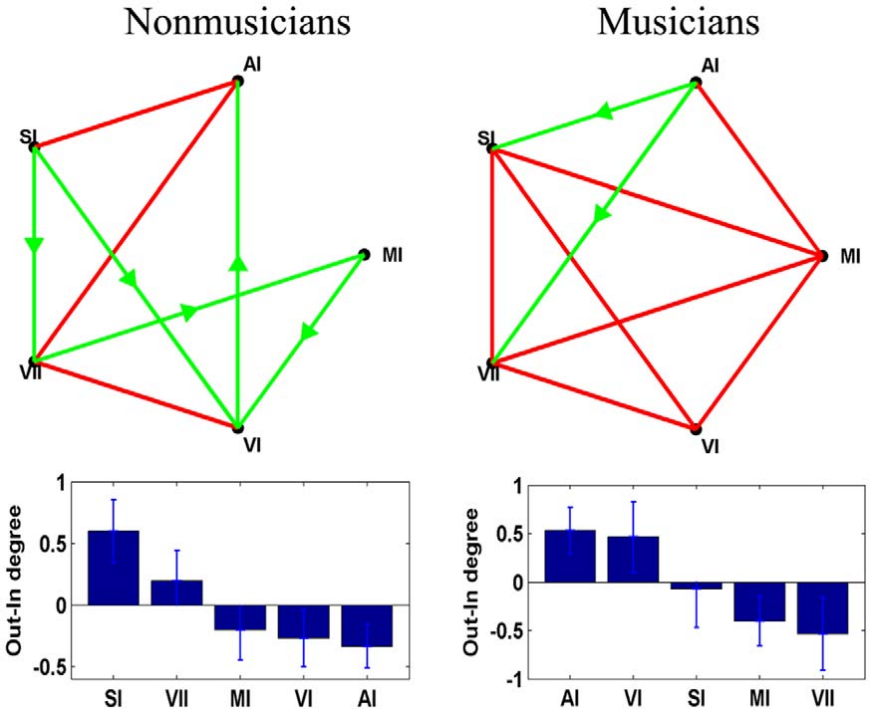
The Granger causality results in musicians(right) and non-musicians(left) displayed separately. The lines without arrows represent bi-directional connections, and the lines with arrow represent uni-directional connections. The group averaged outflow-inflow degree (Out-In degree ) of the nodes in each network are displayed in the bottom panels. Vertical bars indicate estimated standard errors.

The averaged magnitude values of Granger-causality interactions of each pair of ROIs in musicians and non-musicians are shown in Tables 2 and 3 respectively. Figure 4 shows the CGC causal networks of statistically significant Granger-causality interactions in both the musician and non-musician groups. The red lines represent bi-directional connectivity, while the green arrow represents uni-directional connectivity. The group averaged inflow-degree, outflow-degree and outflow-inflow degree of the nodes in the CGC causal connectivity networks were calculated and demonstrated in Table S1 (means and standard errors of these properties) for each group. The mean outflow-inflow degree for each node (and their standard errors) in two groups is shown at the bottom of Fig. 4. The main forward causal interaction (output) in the musician group was from AI and VI to other nodes, whereas it was from SI and VII in the non-musicians. For the causal inflow, AI and VI were dominant nodes in non-musicians and the MI and VII in musicians. To quantify the difference of outflow-inflow degree between the two groups the Wilcoxon signed rank test was used, and a significant difference was found at AI (P = 0.0078). These results suggest that effective connectivity in the motor and perceptual systems exhibited functional changes related to musical training and that the auditory cortex might play a critical role in the functional plasticity exhibited by musicians.

**Table 2:** The averaged values of magnitudes of Granger-causality interaction in musicians.

Origin						
		MI	AI	SI	VII	VI
	**MI**		0.0306±0.011*	0.0396±0.012*	0.0190±0.006*	0.0345±0.008*
	**AI**	0.0260±0.010*		0.0109±0.006	0.0170±0.009	0.0171±0.009
**Target**	**SI**	0.0233±0.008*	0.0157±0.005*		0.0219±0.007*	0.0202±0.007*
	**VII**	0.0278±0.011*	0.0255±0.006*	0.0259±0.009*		0.0414±0.017*
	**VI**	0.0196±0.006*	0.0068±0.004	0.0178±0.006*	0.0289±0.014*	

Note: The rows represent ‘targets’, while the columns represent ‘origins’. * P<0.05 Granger causality interaction. Values of these properties reported are means ± standard errors across subjects.

**Table 3:** The averaged values of magnitudes of Granger-causality interaction in non-musicians.

Origin						
		MI	AI	SI	VII	VI
	**MI**		0.0107±0.005	0.0135±0.007	0.0212±0.006*	0.0237±0.011
	**AI**	0.0097±0.005		0.0420±0.013*	0.0193±0.006*	0.0270*±0.009
**Target**	**SI**	0.0147±0.008	0.0297±0.012*		0.0142±0.008	0.0076±0.004
	**VII**	0.0153±0.009	0.0209±0.009*	0.0160±0.004*		0.0233±0.009*
	**VI**	0.0293±0.011*	0.0252±0.011	0.0341±0.010*	0.0366±0.013*	

Note: The rows represent ‘targets’, while the columns represent 'origins '. * P<0.05 Granger causality interaction. Values of these properties reported are means ± standard errors across subjects.

## Discussion

This study used resting-state fMRI to examine functional and effective connectivity among the motor and multi-sensory cortices in musicians with long-term musical training. The data revealed two main findings in support of our original hypotheses. First, using an ROI-based functional connectivity analysis, we found significantly increased functional connectivity among the motor and multi-sensory cortices in musicians compared with non-musicians. We speculate that enhanced functional integration among the lower-level perceptual and motor networks (relative to the higher-order cognitive networks, e.g. the attention network; DMN) was correlated with improvements in musically-relevant motor and auditory skills. The GCA method was also used to analyze resting state fMRI data to explore effective connectivity differences in the motor and multi-sensory cortices. Our results demonstrated a significant difference of the degree of outflow to inflow at AI between the two groups, and AI was the main forward causal interaction (output) in the musician group. They suggest that the auditory cortex might play an important role in effective connection pattern changes related to long-term music training. Previous studies have reported neural plasticity changes induced by music training using structural imaging [5], [6] and task-based approaches [10], [11], [35]. However, to the best of our knowledge this is the first evidence of spontaneous functional and effective connectivity using resting-state fMRI, supporting the notion that musicians exhibit functional plasticity in motor and perceptual networks.

Previous resting-state fMRI studies have reliably identified intrinsic functional connectivity patterns in the motor and perceptual systems using independent component analysis and functional connectivity analysis [13]–[15], [17]. Coherent oscillatory patterns during rest in RSNs are thought to be involved in the consolidation of past events and preparation for future responses to stimuli [37]. Investigations into the resting state of the brain may provide more insight into the fundamental architectural properties of the brain and structural dysfunction in brain disorders. It has recently suggested that resting-state patterns may also be affected in mental disorders, such as epilepsy [38]–[40] and schizophrenia [41]. In addition, resting state fMRI has been used to examine developmental changes in functional brain organization [42], [43]. The plasticity that characterizes the development of brain systems has been investigated in large-scale networks [44], including those involved in attention and cognitive control [45], and the default mode network [42], as well as in functional connectivity of the anterior cingulate cortex [46]. In the current study, we sought to identify plastic changes in the perceptual and motor networks using a resting-state fMRI dataset in non-musicians and musicians. Besides, we also found a negative correlation pattern with the auditory, somatosensory and visual cortices, which was consistent with the findings of Tian et al's study [15].

Trained musicians have been found to develop tight neuronal coupling of auditory, somatosensory, and motor brain areas. Obviously, the main difference between the professional musicians and non-musicians is the daily intensive practice of playing a musical instrument throughout their lifetime. For example, when a pianist plays a new piece of music, they will typically read the musical notation by eye while playing the keys with their fingers and listening by ear for feedback regulation. As mentioned above, the performance of music involves not only brain motor areas but also somatosensory, auditory, visual, emotional and memory loops [1]–[3], [9], [35], [47]. Our finding of increased spontaneous coherent oscillatory connectivity in perceptual and motor systems is consistent with several previous results from task-based approaches [10], [35]. Our functional connectivity findings might reflect functional consolidation resulting from long-term musical training which requires multi-sensory and motor functional integration. An additional finding of interest was that the visual cortex appeared to be critical in increasing the integration of low-level functional systems in musicians. This finding has not been reported in previous task-based studies and may possibly reflect the use of resting state fMRI to derive changes in intrinsic functional architecture of brain function [12]. Indeed, in previous investigations of brain functional plasticity in musicians, the task-based approaches adopted made extensive use of audiovisual stimuli which may have obscured differences in functional integration between the visual cortex and other sensory cortices in musicians and non-musicians.

The functional connectivity analysis also revealed significantly increased connectivity between the MI and the thalamus in musicians. The human brain has projections from the thalamus to the motor and primary motor cortex, which might contribute to motor-related function, such as voluntary motor control of limbs [48]. Recently, Krause et al. reported stronger interactions between the premotor cortex and thalamus in musicians during an auditory synchronization task, speculating that their observation was related to musical expertise or precise timing in musicians [49]. Our finding also supports the occurrence of functional plasticity changes between the thalamus and motor cortex in musicians at the level of spontaneous oscillations. The present results also revealed increased connectivity between the AI and the cerebellum in musicians. The cerebellum is traditionally considered to be involved in motor coordination, motor skill learning, and various aspects of cognitive and sensory discrimination [50]. A significant difference in cerebellar volume has been reported between musicians and non-musicians, possibly reflecting structural adaptation to long-term motor and cognitive functional demands in the cerebellum during musical training [5]. However, no changes in the connection between the AI and MI were found in the current study. As such, we speculate that the increased connection between AI and cerebellum may implicate cross-modal linkage between the motor and auditory systems. These increased links (MI-thalamus, AI-cerebellum) were not located between the perceptual systems of the cerebral cortex that were the primary focus of our study, but rather reflect motor functional plasticity related to musical training.

In a previous study of directional connectivity among RSNs, it has been reported that the intrinsic activity of low level perceptual and motor networks is strongly intra-dependent in healthy controls [51]. In the present study, the results revealed the apparent circuit of effective connectivity in perceptional and motor networks in both musician and non-musician groups. These results were consistent with the previous observation. Furthermore, the degree of outflow-inflow connections in the auditory node is significantly different between two groups; it was the main output node of information in the effective connectivity pattern of musicians, while it was node with maximum level of inputs in the non-musician group. The auditory network might therefore play a major causal role in plasticity changes in musicians. It is possible that the main causal output node or the driver of causal flow changed to the auditory cortex in musicians because of the importance of auditory cortex for musicians. This finding provides new evidence for auditory plasticity related to musical training.

Several considerations regarding the methodology used in this study should be noted. First, the musician group primarily consisted of females (14/15) and therefore our findings might reflect particular features of female musicians. Some studies have reported gender differences in functional plasticity related to the musical training [52] and therefore potential effects of gender on neural connectivity in musicians should be considered and investigated in the future. Second, lateralized seeds were used in this study and although early studies reported that music was predominantly a right-hemisphere-dominated activity [53], this is now considered to be an oversimplification. For example, memorizing the lyrics of songs has been found to reduce activity in the left hemisphere, while the perception of the violations of expected notes is governed by the right hemisphere [54]. However, it is now known that music listening and performing engage many bilateral brain regions [55]. Because the lateralization of music effects is uncertain, the use of lateralized seeds may have been unsuitable. However, we additionally performed the same functional connectivity analysis seeded at the contralateral ROIs and found that the main result (enhanced functional integration among the lower-level perceptual and motor networks) was the same. This finding also indicates that the functional plasticity in perceptual and motor networks is not lateralized in musicians. The third consideration concerns global signal removal. Global signals that are ubiquitously presented across gray matter can obscure underlying neuroanatomical relationships [56]. These signals should be removed by global (whole brain) signal correction. However, global signal correction as a pre-processing step could introduce negative correlations and reduce positive ones [57] and thus the removal of global signal remains a problem. Third, the relevance of Granger Causality analysis at the neuronal level in resting-state brain networks is not fully understood. Although a few resting-state fMRI studies have revealed a causal influence among the resting-state networks [19], [20], it is believed that this is exerted on the specific brain regions with which other regions interact. Although, the present study focused on the causal relationship among the motor and perceptional networks, the other network (or region) might effect the effective connections among them. However, the mechanism is unclear. We will consider the issues in the future study. We considered the causality analysis as a means to uncover the interaction between the perceptual and motor these networks and the findings were considered together with the results of the correlation analysis, interpreted in terms of brain functional plasticity. Finally, there are two available approaches to process the time series before the GCA evolution in a group of subjects. One involved obtaining separated effect estimate per subject at each pair of ROIs and then combining these at a second-level test for effects across subjects [20], [26], [27], which was used in the current study. The other was the GCA evolution for the concatenated time series from the same ROI across subjects [58]–[60]. Though these approaches were adopted in the variable studies, any weakness still exists respectively. For example, the former did not account for differences in intra-subject variability, and using the letter approach, temporal discontinuities and phase lag were introduced to affect the estimation of causality [59], [60], besides the inter-subject variability was also ignored. The variable approach might lead to the different findings, and the results should be treated with caution.

In conclusion, our results have provided further evidence that intensive musical training exerts a profound influence on fundamental aspects of human brain function. Using resting state fMRI recording, we found significantly increased functional connectivity among the motor and multi-sensory cortices in musicians. These findings indicate enhanced functional integration among the lower-level perceptual and motor networks in musicians, and might reflect functional consolidation (plasticity) resulting from long-term musical training, involving multi-sensory and motor functional integration. In addition, the results indicate that the effective connectivity changes in the auditory cortices might play important roles in functional plasticity in musicians' brains. Thus, the present study has provided novel evidence of spontaneous functional and effective connectivity using resting-state fMRI, lending further support to the notion of functional plasticity in perceptual and motor networks in musicians.

## Materials and Methods

### Subjects

Sixteen musicians and nineteen non-musicians participated in the study after providing informed written consent. The experimental protocol was approved by the research ethics review board of the University of Southwest in China. All participants in the musician group were either students of the University of Southwest of China, studying music as their main subject, or were professional musicians who already possessed an academic degree in music. All musicians had received long-term training in playing the piano (6–20 years), and some also had received variable degrees of training in either the Chinese zither or accordion. All non-musicians were students of the University of Southwest of China, and reported that they had never received formal musical training or played any music instrument. All participants in both groups were right-handed and healthy, with normal brain structure, normal hearing and no history of neurological or psychiatric deficits. All individuals were paid for their participation.

### Image acquisition

All subjects were scanned in a 3T Siemens Trio Tim MRI scanner (Siemens, Erlangen, Germany) with an eight-channel-phased array head coil in Key Laboratory of Cognition and Personality of Ministry of Education, University of Southwest, China. The functional images were acquired using 2D gradient echo-planar imaging (EPI) sequences, with the following imaging parameters: thickness: 3 mm (with 1 mm gap), repetition time (TR)  = 2,000 ms, echo time (TE)  =  30 ms, field of view (FOV)  = 22 cm ×22 cm, flip angle  = 90°, matrix  = 64×64. A total of 205 volumes (32 slices per volume) were acquired during 410 seconds. To ensure steady-state longitudinal magnetization, the first five volumes were discarded. During data acquisition, participants were instructed to relax with eyes closed, without falling asleep. Anatomical T1-weighted images were acquired using a three-dimensional (3D)-spoiled gradient recalled (SPGR) sequence, generating 176 axial slices (thickness: 1 mm (no gap), TR = 8.5 ms, TE = 3.4 ms, FOV = 24 cm×24 cm, flip angle  = 12°, matrix = 512×512).

### Data preprocessing analysis

Preprocessing and analysis of fMRI data was carried out using SPM8 software (Statistical Parametric Mapping; http://www.fil.ion.ucl.ac.uk/spm). We conducted slice time correction, 3D motion detection and correction, spatial normalization to the MNI template supplied by SPM, and spatial smoothing using an isotropic Gaussian kernel (8 mm full width at half maximum). Data were excluded if head motion exceeded 1 mm and 1 degree during fMRI acquisition. As in our previous study [61], three procedures were processed using in-house software to remove possible spurious variance from the time series at each voxel. (i) Temporal band-pass filtering (pass band 0.01–0.08 Hz) was conducted using a phase-insensitive filter, which was performed to reduce the effects of low-frequency drift and high-frequency noise (ii) The time series was further corrected for the effect of six head motion parameters obtained in the realigning step, and the effect of the signals from a cerebrospinal fluid (CSF) region, a white matter (WM) region and averaged signals from the whole brain.

### Functional connectivity analysis

We examined the RSNs of the motor cortex and three representative perceptual cortices, namely, the auditory, somatosensory and visual cortices, using a region of interest (ROI)-based functional connectivity analysis. Based on previous research work [15], [36], four spherical regions (radius 10 mm) were selected as seeds to identify four different networks; the right primary motor cortex ( MI, Talairach coordinates [42,−21,54]) for the motor network, left primary auditory cortex (AI, Talairach coordinates [−56,−17,6]) in the auditory network, left primary somatosensory cortex (SI Talairach coordinates [−47,−31,55]) in the somatosensory network, and left V2 area (VII,Talairach coordinates [−27,−84,−2]) in the visual network. Furthermore, the left primary visual ROI (VI, Talairach coordinates [−9,−84,6]) was also selected for the visual network. The mean BOLD signal intensity time series was extracted from the five ROIs. Subsequently, functional connectivity analysis was performed between the seed and all voxels in the brain data. Individual correlation coefficients were normalized to z-scores using Fisher's r-to-z transformation.

SPM8 was used to assess voxel-wise statistical significance of functional connectivity at the group level and differences between groups. First, an individual z-score map was used in a random effects one-sample t-test. A statistical map of significant functional connectivity of each seed for each group was acquired. Next, to examine the difference of functional connectivity between musicians and non-musicians in the five networks, the z-score maps were also processed in a random effects two-sample t-test embedded in SPM8 in the mask of each perceptual and motor network. The mask was created based on the conjunction of two maps which were significantly positively correlated with the seed in the respective group. The mask was only used to determine the area of the comparison between groups, so the loose statistical threshold p<0.05 was adopted.

### Granger causality analysis

Effective connectivity was based on GCA [25], which holds that a time series X(t) may “Granger cause” another one time series Y(t), if information about the past of X(t) helps to predict the time series Y(t), better than knowing the past of Y(t) alone [62]. Another important extension of Granger's original definition of causality is the consideration of the multivariate case: For three or more simultaneous time series, the causal relation between any two of the series may be direct, mediated by a third one, or a combination of both. Recently, conditional Granger causality analysis (CGCA) [63] has been proposed to estimate functional coupling effectively in multivariate data sets [19], [26]. In the current study, CGCA was performed to characterize the effective connectivity among the brain regions in perceptional and motor functional systems using the Granger Causal Connectivity Toolbox [64]. Consider the case of three time series X(t), Y(t), and Z(t). First, the joint autoregressive representation for X(t) and Z(t) can be written as
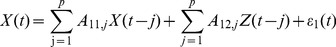
(1)

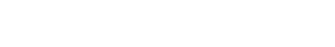
and the noise covariance matrix can be represented as



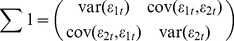
(2)Next, we consider the joint autoregressive representation for a system involving all the three time series X(t), Y(t), and Z(t) as
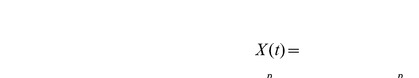


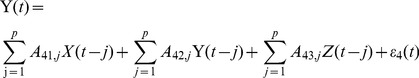
(3)

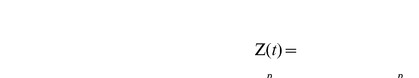
and the noise covariance matrix for the above system can be represented as



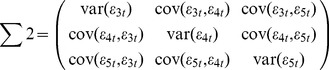
(4)where p is the order of the autoregressive model; and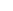, i = 1,. . . . . ., 5 are the prediction error, which are uncorrelated over time. From these two sets of equations, we define the conditional Granger causality from time series Y(t) to X(t) conditional on time series Z(t) as



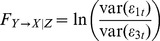
(5)It is worth pointing out that Eq. 5 is essentially a log likelihood ratio test, comparing models with and without the directed connection from Y(t) to X(t).When the causal influence from time series Y(t) to X(t) is entirely mediated by other time series Z(t), the coefficients 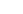 in Eq. 3 are uniformly zero, and 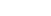  =  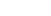. As such, 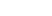  = 0, meaning that no further improvement in the predication of time series X(t) can be expected by including past measurements of time series Y(t) conditioned on the other time series Z(t). On the contrary, when a direct influence from time series Y(t) to X(t) exists, the inclusion of past measurements of time series Y(t) in addition to that of time series X(t) and Z(t) should result in better predictions of time series X(t), leading to 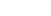
>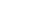, and 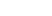
>0 [65].

First, for each subject, conditional Granger causality was assessed for the preprocessed time series form each ROI pair (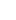 = 20) separately. The time series of one of the network can be associated with X(t), and another one with Y(t). Z(t) represents all the remaining three ones. The time series of all predefined ROIs were simultaneously modeled using the first order multivariate regression (MVR). This order of the autoregressive model used for scomputation of the influence measure was selected using the Bayesian information criterion. The optimal order is almost always equal to 1 (more than 90% of the times) for all subjects, so we stick to this value of 1.

The G-causality interaction coefficients can be established via an F-test implemented in the Granger Causal Connectivity Toolbox [64] on the null hypothesis that conditional Granger causality is zero [25]. For each subject, dominant connections that passed a p = 0.05(FDR-corrected) significance level were used for the following analysis. To describe the causal flow at a within-group level, a causal connectivity graph within-group was constructed with those connections for which the G-causality is significantly different from the null distribution (wilcoxon signed rank test, P<0.05). Since the null distribution of the G-causality was unknown, the framework of the bootstrap methodology were used to obtain the null distribution [66]. This approach was widely employed in many fMRI studies to find the level of statistical significance without making assumptions on the underlying distribution of the data [20], [23], [26]. By choosing random samples with replacement from a data set x and y a large number (the number was set to be 1000 in the present study) of surrogate time series are generated whose dynamic and statistical properties are similar with that of the original. Computation of the G-causality values over these surrogates gives a bootstrap empirical distribution that characterizes the null hypothesis of no influence between time series x and y. Finally, the inflow-degree and outflow-degree of the nodes in the CGC causal connectivity networks were calculated for each subject to evaluate the causal inflow/outflow connections. The outflow-degree was determined by the number of significant causal afferent connections from a node in the network to any other nodes. Similarly, the inflow-degree was determined by the number of significant causal efferent connections from a node in the network to any other nodes. The outflow-inflow degree was represented by the difference between the inflow- and outflow-degrees.

## Supporting Information

Table S1 **Degree of Network analysis in the Granger casual influence.** Outflow: Number of Granger causal afferent connections from a node in the network to any other node. Inflow: Number of Granger causal efferent connections from a node in the network to any other node. Outflow-Inflow: Difference between outflow-degree and inflow-degree is a measure of the causal flow a node in the Granger causality network. Values of these properties reported are means ± standard errors across subjects.
(DOC)
