# Supplementary material for: Musical Training Induces Functional Plasticity in Perceptual and Motor Networks: Insights from Resting-State fMRI
Source: PLoS One. 2012 May 7;7(5):e36568. doi: 10.1371/journal.pone.0036568 (PMC3346725; doi:10.1371/journal.pone.0036568)
Supplement: Table S1 — Degree of Network analysis in the Granger casual influence. Outflow: Number of Granger causal afferent connections from a node in the network to any other node. Inflow: Number of Granger causal efferent connections from a node in the network to any other node. Outflow-Inflow: Difference between outflow-degree and inflow-degree is a measure of the causal flow a node in the Granger causality network. Values of these properties reported are means ± standard errors across subjects. (DOC) [file pone.0036568.s001.doc]

**Supplementary Table:**

Degree of Network analysis in the Granger casual influence

|  | Non-Musicians | | | Musicians | | |
| --- | --- | --- | --- | --- | --- | --- |
|  | Outflow | Inflow | Outflow-Inflow | Outflow | Inflow | Outflow-Inflow |
| MI | 1.13±0.29 | 1.33±0.28 | -0.20±0.24 | 1.60±0.21 | 2.00±0.23 | -0.40±0.25 |
| AI | 1.33±0.20 | 1.66±0.20 | -0.33±0.17 | 1.66±0.24 | 1.13±0.26 | 0.53±0.23 |
| SI | 1.80±0.27 | 1.20±0.17 | 0.60±0.27 | 1.67±0.29 | 1.73±0.26 | -0.06±0.40 |
| VI | 1.73±0.22 | 1.53±0.23 | 0.20±0.24 | 1.47±0.23 | 2.00±0.25 | -0.53±0.37 |
| VII | 1.40±0.23 | 1.66±0.29 | -0.26±0.23 | 1.86±0.26 | 1.40±0.22 | 0.46±0.36 |

Outflow: Number of Granger causal afferent connections from a node in the network to any other node.

Inflow: Number of Granger causal efferent connections from a node in the network to any other node.

Outflow-Inflow: Difference between outflow-degree and inflow-degree is a measure of the causal flow a node in the Granger causality network.

Values of these properties reported are means ± standard errors across subjects.
